# Food Processing and Maillard Reaction Products: Effect on Human Health and Nutrition

**DOI:** 10.1155/2015/526762

**Published:** 2015-01-08

**Authors:** Nahid Tamanna, Niaz Mahmood

**Affiliations:** ^1^Graduate Program in Biological Sciences, University of Manitoba, Winnipeg, MB, Canada R3T 2N2; ^2^Graduate Program in Biochemistry and Medical Genetics, University of Manitoba, Winnipeg, MB, Canada R3E 0J9

## Abstract

Maillard reaction produces flavour and aroma during cooking process; and it is used almost everywhere from the baking industry to our day to day life to make food tasty. It is often called nonenzymatic browning reaction since it takes place in the absence of enzyme. When foods are being processed or cooked at high temperature, chemical reaction between amino acids and reducing sugars leads to the formation of Maillard reaction products (MRPs). Depending on the way the food is being processed, both beneficial and toxic MRPs can be produced. Therefore, there is a need to understand the different types of MRPs and their positive or negative health effects. In this review we have summarized how food processing effects MRP formation in some of the very common foods.

## 1. Introduction

**Figure 1:**
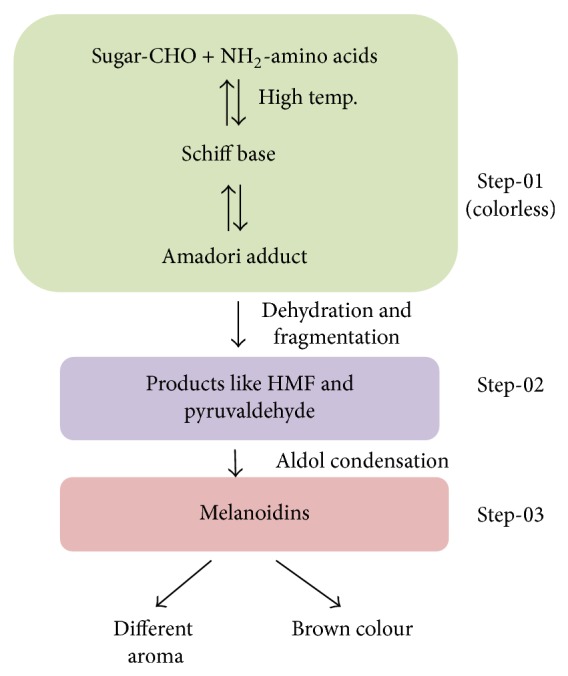
Schematic representation of “Maillard reaction” and flavour formation in food.

The Maillard reaction has been named after the French physicist and chemist Louis Camille Maillard (1878–1936) who initially described it. It is often defined as nonenzymatic browning reaction. While foods are processed or cooked at high temperature, a chemical reaction occurs between amino acids and reducing sugars which generate different flavours and brown colour (Figure 1). So it is often used in food industry for giving food different taste, colour, and aroma.

Based on literature, Hodge (1953) first described the steps involved in Maillard reaction products (MRPs), also known as advanced glycation end-products (AGEs), formation. The whole process of MRPs formation can be divided into three major stages depending on colour formation. At the first stage, sugars and amino acid condense, and following condensation, Amadori rearrangement and 1-amino-1deoxy-2 ketose form. In the second stage, dehydration and fragmentation occur in the sugar molecules. Amino acids are also degraded in this stage. Hydroxymethylfurfural (HMF) fission products such as pyruvaldehyde and diacetyl are formed in this intermediate stage. This stage can be slight yellow or colourless. In the final stage, aldol condensation occurs and finally the heterocyclic nitrogenous compounds form, melanoidins, which is highly coloured [1]. Maillard reaction can also take place in living organisms. It has been reported that some MRPs particularly melanoidins have beneficial effects on health such as antioxidative [2] and antibiotic [3] effects. However some reports have also suggested that MRPs such as high carboxymethyl lysine (CML) promote diabetes and cardiovascular diseases while acrylamide acts as a carcinogen [4–6].

There is an ever-increasing preference for instant meal rather than traditional cooking, especially among the new generation of people. It has been reported that people consuming high amount of processed meat, pizza, or snacks develop insulin resistance and metabolic syndrome compared to people having high intake of vegetables and low processed food [7]. MRPs that change during food processing might be one of the important factors for either disease progression or combating diseases. In this review, we have summarized the changes of MRPs which occur during processing of foods.

## 2. Soybean Processing and MRPs Formation

Soybean is widely used as flours, grits, flakes, isolates, concentrates, and textured soya proteins and also as cooking oil. Soybeans play important role in cardiovascular diseases, osteoporosis, and cancer [8]. So processing of soybean is an important factor for maintaining its nutritional quality. Cooking at high temperature may generate MRPs which can be good or bad for health. However, soybean must be processed before consumption. Žilić et al. (2014) assessed the level of furosine, hydroxymethylfurfural (HMF), and acrylamide in soybean during extrusion, microwave, and infrared heating processes [9]. They found that microwave heating for short time (1-2 min) generates high levels of acrylamide, whereas long time heating (3–5 min) generates lower levels of acrylamide. During extrusion and infrared heating, acrylamide formation greatly increased with time and temperature. HMF level increased in all three processes with increased time and temperature and it was significantly higher in microwave treatment. From the beginning of heat treatment, furosine level was higher in the extrusion and infrared treatment whereas in the microwave heating it was increased to maximum value after 3 min but at 4 min this value was similar with 2 min. Their results showed that microwave heating improved the antioxidant properties of soybean by 50% compared to raw soybean [9]. Even though this study has reported that total flavonoids increase at 100°C with the exception of microwave heating which occurs at 45°C, another study showed that when soybean is soaked in water and heated afterwards at 98°C, almost half (44%) of the raw flavonoids were lost in the final product [10]. This might be due to the presence of moisture content since decreasing moisture was shown to be associated with elevated levels of MRPs in the extrusion and infrared heat treatment [9].

## 3. MRPs on Milk Processing

Milk is a beverage that is consumed throughout the world. At present, a large percentage of the milk consumed by people, especially in the western countries, is processed rather than raw milk. Ultra high temperature (UHT) treatment or conventional sterilization process is often used to process milk for improving quality and safety. Milk is rich in protein and sugar. So it is obvious that processing of milk at high temperature may lead to the formation of MRPs. Several methods have been approached to determine the extent of MRPs during milk processing. Both initial and advanced staged MRPs have been used as indicators of browning reaction which occurred in milk [11, 12]. MRP formation affects protein and mineral bioavailability greatly. In the early stages, lactose in milk blocks the amino group of lysine to form the Amadori product called lactulosyllysine which changes the protein bioavailability [13]. It is also known that MRPs may behave as chelating agents to chelate metal cations by forming different soluble and insoluble complexes and thereby can affect mineral bioavailability [14]. So processing of milk by heat-treatment requires attention, especially for the infants, as milk is the sole source of nutrients at that stage of life.

It has been reported that conventional sterilized bottle milk has different chemical composition compared to the UHT treated milk [15]. HMF level is often used to assess the progression of MRPs formation. However, milk processed at UHT may have different levels of HMF [16] due to the presence of some other factors such as vitamin A, casein, and iron [17]. So during milk processing, along with heat treatment, other relevant factors should be considered for preserving the nutritious value. A recent study has shown that use of enzymes like Faox I and Faox II might inhibit Maillard reaction development [18].

## 4. Pasta Processing and MRPs

Pasta is considered one of the healthy foods suited to balanced diet; and its consumption is becoming increasingly high throughout the world. Pasta has different varieties and shapes. Till 2009, 310 different types of both dried and fresh pasta have been documented [19]. Although an Italian law exists for making high quality pasta [20], different steps of pasta processing cycle such as mixing, kneading, shaping, and drying can affect the pasta quality. Among these four steps, drying is most important for the quality of the product. For traditional drying methods, low temperatures (29 to 40°C) are required but it takes 24 to 60 hours to complete the process. So for time management and to increase the production rate, high-temperature short-time drying processes have been widely preferred. While pasta is processed at high temperature, it brings benefits in terms of productivity, cost, colour intensity, and nutritious value [21]. Moreover, application of high drying temperatures in pasta increases firmness and decreases stickiness [22]. Besides these beneficial effects, high heat treatment leads to the formation of MRPs that ultimately can change flavor, color, functional properties, and nutritional value [23]. Furosine is widely used as pasta quality assessment. It has been reported that pasta processed at high temperature had decreased level of total carotenoid level [24]. Along with furosine, maltulose is also proposed as a maker for pasta quality assessment [25].

## 5. Meat Processing and MRPs

MRPs like heterocyclic amine (HCA) level increases with elevated cooking temperature; and this phenomenon is more pronounced in meat than fish [26]. Meat is cooked at high temperature either by frying, roasting, and boiling or in oven. While positive correlations have been found between the intakes of HCAs from foods and increased risk of various types of human cancer [27–29], some other studies have not found any correlation between HCAs and cancer risk [30–32]. Several studies have demonstrated that processes like frying and broiling can cause the formation of high amounts of HCAs [27, 33–35]. On contrary, these HCAs produce different flavour and tastes in foods. Heterocyclic compounds such as pyrazine, oxazole, and thiazoles are primarily responsible for forming flavour in roasted compound. During high heat treatment and grilling process, pyrazines level significantly increased [36]. It is suggested that the alkylpyrizne is formed via the condensation of two alpha amino ketone molecules derived from the Strecker degradation [37], which is an intermediate of Maillard reaction pathway.

In processed food, more than 25 types of heterocyclic amines (HCA) have been identified [38]. A study has shown that when duck meat was cooked by charcoal grilling, deep-frying, roasting, microwave cooking, pan frying, or boiling, MRPs were higher in the pan frying process compared to the other four methods of cooking. Liao et al. (2012) [39] reported that boiling and microwave cooking were the most appropriate methods to process duck meat in terms of MRPs formation. However in another study, it has been found that both charcoal grilled duck and chicken breast had high level of HCAs compared to pan fried meat. They found that roasting decreases HCAs significantly [40].

In another study, beef steak and hamburger patties were processed by pan-frying, oven-broiled, and grilled or barbecued to four levels of doneness (rare, medium, well cooked, or very well cooked) [41]. Beef roasts were processed on oven by rare, medium, and well cooking. They measured five different HCAs. The level of 2-amino-3,4-dimethylimidazo[4,5-*f*] quinoline was higher in well-cooked steak and hamburger patties. Like duck and chicken roast, roasting beef did not contain any of the 5 HCAs, but the gravy made from the drippings from well-done roasts had two types of HCAs [41]. From the three different studies, it can be suggested that roasting of meat (chicken, duck, and beef) generates less amount of HCAs compared to other methods.

In recent days, people consume more ready-to-eat food due to lack of time. Puangsombat et al. (2011) assessed HCAs level in some ready-to-eat products. They found that HCAs were higher in rotisserie chicken skin. In the other assessed food, HCA level was found in the order as follows: rotisserie chicken meat, deli meat products, and pepperoni [42]. However, it has been reported that commercially cooked meats and restaurant meats contain low amounts of HCAs [43, 44].

## 6. Coffee Bean Processing and MRPs

As a beverage, coffee is an important item in the lives of billions of people and is also one of the most traded food products in the world. The brewed coffee has emerged as the second most consumed drink after water [45, 46]. The desirable fragrance of coffee beverages develops during roasting procedures. Typical roasting temperatures range from 180 to 250°C, and roasting time varies between 2 and 25 min depending on the process employed [47]. During roasting procedures, internal temperature exceeds 180°C which results in the occurrence of Maillard reaction, carbohydrate caramelization, and pyrolysis of organic compounds [47]. Maillard reaction generates melanoidins upon roasting, which accounts for 29% of the dry weight of brewed coffee bean [47]. Coffee melanoidins are formed via polymerization reactions of furans and/or pyrroles, during the advanced stages of the Maillard reaction, and linked by poorly defined polycondensation reactions [48]. Even though roasting decreases coffee bean carbohydrates, protein, and lipid level, the level of caffeine remains relatively stable on roasting. MRPs, caffeine, nicotinic acid, and some other components of coffee bean protect teeth from* Streptococcus mutans* which is considered to be the major causative agent of dental caries in humans [49].

## 7. Plant Derived Food Processing and MRPs

Consumption of diets rich in fruits and vegetables renders many health benefits to us. However, processing method plays an important role in dictating the magnitude of the beneficial health effects obtained from fruits and vegetables. Depending on treatment temperature, furoylmethyl derivatives (FM) have been found in processed vegetables and fruits like orange juices [50] and processed tomato products [51] and also in dehydrated carrots [52]. It has been shown that dehydrated carrot contains significantly high amount of FM compared to carrot juices, baby carrot, or tinned carrot. It is suggested that processing time during the heat treatment plays an important role for FM formation [52]. Dueik and Bouchon (2011) have reported that, by vacuum frying of carrot chips, potato and apple slices can help to retain their total carotenoids and ascorbic acid levels significantly [53].

When vegetables are treated at low temperature, prooxidants are generated, whereas treating at high temperature decreases the prooxidants and increases antioxidant properties due to the production of MRPs [54]. Such antioxidant activity of the MRPs comes from the high molecular weight brown compounds that are formed in the advanced stages of the reaction [54]. However, it should be mentioned here that MRPs can also exhibit prooxidant properties [55, 56].

MRPs can prevent the enzymatic browning reaction caused by polyphenol oxidase (PPO) [57]. Plant derived products, such as fruits and vegetables, produce many endogenous phenolic compounds during postharvest handling and processing. These compounds are oxidized by oxidoreductase enzymes like polyphenoloxidases (PPOs) and tyrosinases. This reaction, in turn, generates highly reactive quinonic compounds that are condensed and polymerized to produce brown pigments and thereby decreases the quality of the food product. MRPs can prevent this enzymatic process at the initial step of this reaction and thereby help to maintain the product quality. Besides antibrowning, MRPs has also been shown to render antiallergenic property for cherry derived allergens [58].

## 8. Some Other Impacts of MRP-Derived Food

Angiotensin-I converting enzyme (ACE) is the regulatory enzyme for upregulation of blood pressure. ACE inhibitory peptide lowers blood pressure by inhibiting ACE enzyme [59]. Rufián-Henares and Morales (2007) have demonstrated that the melanoidins isolated from seven amino acid-glucose model systems were all shown to cause inhibition of ACE* in vitro* [60]. Recently, Hong and colleagues (2014) have shown that, under the appropriate conditions, Maillard reaction can effectively improve the ACE inhibitory activity of casein hydrolysate [61].

It has been claimed that administration of a Maillard browning reaction product obtained from an extract of* Panax* species plant comprising ginsenoside Re or ginsenoside-derived saccharide treated with amino acid at temperatures between 100 and 130°C can either prevent, improve, or treat a renal disease [62].

Food derived versatile MRPs can act as bactericidal for a wide number of pathogens. For example, aminoreductone can act as a more effective bactericidal for four* Pseudomonas aeruginosa isolates*, one multidrug-resistant* Pseudomonas aeruginosa* (MDRP), one* Escherichia coli*, one methicillin-susceptible* Staphylococcus aureus*, and one methicillin-resistant* Staphylococcus aureus* (MRSA) compared to mikacin, ciprofloxacin, imipenem, and levofloxacin [63]. MRPs have also been shown to be effective against yeast [64].

## 9. Conclusion and Perspectives

Maillard reaction products have both positive and negative impacts on health. Diverse MRPs act as antioxidants, bactericidal, antiallergenic, antibrowning, prooxidants, and carcinogens. Most of these properties depend on processing of food. High temperature heating makes some food nutritious, whereas some of the foods lose their nutritional value. Many strategies are employed in the food industries to reduce the production of MRPs. For example, acrylamide has been classified as a probable carcinogen to humans by the International Agency for Research on Cancer [65]. During food preparation at high temperature, acrylamides are formed in many types of foods via Maillard reaction [66–68]. To reduce the amount of acrylamide, asparaginase has been successfully used in laboratory for potatoes and cereals [69, 70]. It has also been reported that injection of CO_2_ during extrusion process helps to reduce the level of acrylamide [71].

This review was aimed at summarizing our current knowledge regarding the changes in food mediated by Maillard reaction during the food processing steps. This may provide useful insights for those related to food processing facilities.
